# Clinical Outcomes of Injectable Hyaluronic Acid-Glucosamine Combination in Knee Osteoarthritis: A Saudi Multicenter Retrospective Study

**DOI:** 10.7759/cureus.111228

**Published:** 2026-06-21

**Authors:** Amr M Ali, Yaser A Allam, Mohsen A Ali

**Affiliations:** 1 Orthopaedic Surgery, Sultan Bin Abdulaziz Humanitarian City (SBAHC), Riyadh, SAU; 2 Orthopedic Surgery, New Jeddah Clinic Hospital, Jeddah, SAU; 3 Computer and Communication Engineering, Faculty of Engineering, Alexandria University, Alexandria, EGY

**Keywords:** glucosamine, hyaluronic acid, intra-articular injection, knee osteoarthritis, knee pain, lequesne index, orthopedic outcomes, retrospective study, saudi arabia, viscosupplementation

## Abstract

Background

Knee osteoarthritis (OA) remains one of the most common issues causing chronic pain, reduced mobility, and functional limitations in everyday orthopedic practice. Although hyaluronic acid injections are often used for managing symptoms, clinical data regarding injectable hyaluronic acid-glucosamine combination products remain scarce. In this study, we evaluated short- and mid-term clinical outcomes of a single intra-articular hyaluronic acid-glucosamine injection in patients with symptomatic knee OA treated in routine outpatient settings.

Methods

This multicenter retrospective study included 44 patients with symptomatic knee OA treated in orthopedic outpatient clinics in Riyadh and Jeddah, Saudi Arabia. During routine care, each patient received a single, clinically guided, intra-articular hyaluronic acid-glucosamine injection. Outcomes were assessed using the Lequesne Algofunctional Index and visual analog scale (VAS) pain scores recorded at baseline, approximately six weeks, and six months post-injection. Secondary outcomes included patient satisfaction and treatment-related adverse events.

Results

Mean Lequesne score decreased from 19.18 ± 3.41 at baseline to 16.36 ± 3.63 at approximately six weeks and 13.28 ± 3.09 at six months based on paired t-test analysis (p < 0.001). Mean VAS pain score decreased from 7.48 ± 1.17 at baseline to 5.73 ± 1.53 at six weeks and 4.21 ± 1.20 at six months (p < 0.001). Most patients reported favorable symptomatic and functional improvement, with an overall mean satisfaction score of 4.21 ± 0.73 out of 5. Reported adverse events were mild and transient, consisting mainly of short-duration post-injection pain and minor local inflammatory reactions. No serious treatment-related adverse events or joint infections were documented.

Conclusions

In this retrospective cohort, a single intra-articular hyaluronic acid-glucosamine injection was associated with improved pain and functional scores over a six-month follow-up period in patients with symptomatic knee OA. The intervention was generally well tolerated in this routine outpatient cohort. These findings should be interpreted cautiously, given the small sample size and absence of a comparator group.

## Introduction

Knee osteoarthritis (OA) is one of the most common causes of chronic knee pain and functional limitation in orthopedic practice. Although it is often discussed as a degenerative joint disease, the clinical problem is usually broader than the radiograph. Patients commonly present with pain during walking, stiffness after rest, difficulty using stairs, reduced walking distance, and gradual loss of confidence in daily movement. These symptoms become especially relevant in older adults and in patients with obesity, where knee OA may significantly affect independence and quality of life [1].

Treatment is usually approached in a stepwise manner. Most patients are initially managed with education, weight reduction, physiotherapy, activity modification, oral analgesics, and nonsteroidal anti-inflammatory drugs when appropriate. In daily outpatient practice, however, many patients continue to report pain and functional restrictions despite these measures. This is particularly common in patients with moderate or advanced radiographic disease, high body mass index, bilateral symptoms, or long-standing mechanical complaints. For such patients, intra-articular injections are often considered as part of nonoperative care, especially when the patient is not ready for arthroplasty or prefers to delay surgery [2,3].

Hyaluronic acid has been used for many years as an intra-articular treatment option for symptomatic knee OA. Its clinical use is based on the concept of viscosupplementation, aiming to improve the viscoelastic environment of the osteoarthritic joint rather than acting only as a short-term analgesic injection. The published evidence remains mixed. Some recent reviews have questioned the magnitude and consistency of benefit compared with placebo, while others have reported clinically relevant improvement in pain after intra-articular hyaluronic acid injection [4,5]. This uncertainty makes it important to report clinical outcomes carefully, without overstating the effect or presenting routine injection therapy as disease-modifying treatment.

Glucosamine is also commonly discussed in OA care, but most of the available evidence relates to oral glucosamine or oral glucosamine-chondroitin combinations. Results across trials and systematic reviews have been variable, and its role remains debated [6,7]. Clinical data are much more limited for injectable preparations that combine hyaluronic acid with glucosamine in a single intra-articular product. This creates a practical evidence gap because such products may be used in real orthopedic clinics even when formal comparative evidence is still developing.

In outpatient settings, outcome measurement also needs to be practical. Very long questionnaires may be difficult to use consistently in busy clinics or during routine follow-up. The Lequesne Algofunctional Index is a short osteoarthritis-specific clinical index that evaluates pain or discomfort, maximum walking distance, and activities of daily living. Its structure makes it suitable for routine documentation because it is focused, clinically understandable, and relatively quick to administer while still capturing the main functional problems that matter to knee OA patients [8].

In Saudi outpatient orthopedic practice, many patients with knee OA present with advanced symptoms, elevated body mass index, and a strong preference for nonoperative treatment when possible. Retrospective clinical data from such settings may provide useful early information about treatment response, tolerability, and patient satisfaction in a population that resembles routine practice rather than a highly selected trial cohort.

The aim of this study was to evaluate short- and mid-term clinical outcomes following a single intra-articular hyaluronic acid-glucosamine injection in patients with symptomatic knee OA treated in two Saudi outpatient orthopedic clinics. Outcomes were assessed using routinely documented Lequesne Algofunctional Index scores, visual analog scale pain scores, patient satisfaction, and adverse event documentation.

## Materials and methods

Study design and setting

This was a retrospective multicenter observational study conducted in two outpatient orthopedic clinics in Saudi Arabia, one in Riyadh and one in Jeddah. The study reviewed routinely documented clinical outcomes of patients with symptomatic knee osteoarthritis who received a single intra-articular hyaluronic acid-glucosamine injection as part of usual outpatient orthopedic care.

Data source and extraction

Data were extracted from the electronic health records of both participating clinics. The study included patients treated between 15 September 2025 and 24 December 2025. Follow-up outcomes were reviewed according to the predefined routine follow-up windows: approximately six weeks after injection within ±1 week and approximately six months after injection within ±2 weeks. No separate case record form was used; extracted variables were organized in a structured study spreadsheet, including demographic characteristics, radiographic grade, injection details, Lequesne score, VAS pain score, satisfaction score, follow-up completion, and adverse events.

Patient selection

Patients were included if they had symptomatic knee osteoarthritis, persistent pain or functional limitation despite previous conservative management, and available baseline and follow-up clinical documentation. Conservative treatment before injection included one or more of the following: physiotherapy, oral analgesics, nonsteroidal anti-inflammatory drugs, activity modification, or other routine nonoperative measures.

Patients were excluded if they had secondary osteoarthritis, inflammatory arthritis, recent knee surgery, recent intra-articular knee injection, active local or systemic infection, or incomplete baseline clinical documentation. Patients with advanced radiographic disease, obesity, varus or valgus deformity, and bilateral osteoarthritis were not excluded, as these features reflect a typical orthopedic outpatient population.

Clinical and radiographic assessment

Baseline data included age, sex, body mass index (BMI), involved side, osteoarthritis pattern, and Kellgren-Lawrence (KL) grade based on standing knee radiographs [9]. Radiographic grading was taken from routine clinical radiology reporting and was not centrally reviewed. In patients with bilateral disease, right and left knee KL grades were recorded separately; however, patient-reported clinical outcomes were analyzed at the patient level rather than as separate knees.

Injection protocol

All patients received a single intra-articular injection of Viscoart Forte GGP (ERC Biyoteknoloji Sanayi ve Dış Ticaret Anonim Şirketi, Pendik, İstanbul, Türkiye), a commercially available hyaluronic acid-based combination product. According to the manufacturer’s product information leaflet, each 3 mL prefilled syringe contains sodium hyaluronate 90 mg, L-proline 45 mg, glycine 60 mg, and N-acetylglucosamine 90 mg [10]. The injection was administered once using a standard clinically guided outpatient technique under routine clinical conditions. A medial or lateral entry point was used according to the treating surgeon’s preference.

Ultrasound guidance was not used. Patients were allowed to continue simple analgesics, nonsteroidal anti-inflammatory drugs when appropriate, and physiotherapy during the follow-up period. Patients who received additional intra-articular knee injections during the follow-up period were not included in the outcome analysis.

Outcome measures

The primary outcome measure was the change in the Lequesne Algofunctional Index score from baseline to follow-up [8]. The Lequesne index was used because it is a short osteoarthritis-specific clinical index that evaluates pain or discomfort, maximum walking distance, and activities of daily living. The total score ranges from 0 to 24, with higher scores indicating greater symptom severity and functional limitation. Commonly used severity categories are 0 for no handicap; 1-4 for mild; 5-7 for moderate; 8-10 for severe; 11-13 for very severe; and ≥14 for extremely severe handicap.

Secondary outcomes included visual analog scale (VAS) pain score, patient satisfaction, and documented adverse events [11].

Clinical outcomes were reviewed at baseline, approximately six weeks, and six months after injection. A practical follow-up window was accepted to reflect routine outpatient scheduling: a six-week follow-up was accepted within ±1 week, and a six-month follow-up was accepted within ±2 weeks.

Safety assessment

Adverse events were identified from routine clinical notes and follow-up documentation. Events of interest included post-injection pain, local redness, swelling, stiffness, infection, inflammatory reaction, and any need for further intervention. Serious adverse events were defined as events requiring hospitalization, urgent surgical intervention, or resulting in major clinical deterioration.

Ethical considerations

This study used anonymized retrospective clinical outcome data collected during routine outpatient orthopedic care. No patient-identifying information, images, or personal details were included in the manuscript. All patients had provided clinical consent for treatment as part of routine care. The participating clinics did not require formal IRB review for this anonymized retrospective record review.

Statistical analysis

Continuous variables were reported as mean ± standard deviation (SD). Categorical variables were reported as frequencies and percentages. Paired t-tests were used to compare baseline Lequesne and VAS scores with follow-up values at approximately six weeks and six months. Patients with missing six-month outcomes were excluded only from the six-month paired analysis. A descriptive comparison was performed between patients who completed the six-month follow-up and those who were lost to six-month follow-up. An exploratory descriptive subgroup analysis was also performed according to the worst recorded KL grade per patient, comparing patients with KL grade 4 involvement against those with KL grade 2 or 3 involvement. Because of the limited sample size, these additional comparisons were interpreted descriptively. A p-value of <0.05 was considered statistically significant.

## Results

A total of 44 patients with symptomatic knee osteoarthritis were included in the final analysis. The cohort reflected a routine outpatient orthopedic population, with a predominance of female patients, elevated body mass index, and frequent bilateral knee involvement. Radiographic disease severity was also clinically relevant, as many patients had Kellgren-Lawrence grade 3 or 4 osteoarthritis at baseline. The main demographic and clinical characteristics of the study cohort are summarized in Table 1.

**Table 1:** Baseline Demographic and Clinical Characteristics Values are presented as mean ± standard deviation or number (%). BMI: body mass index; OA: osteoarthritis; KL: Kellgren-Lawrence; VAS: visual analog scale. KL grade involvement indicates patients with that grade recorded in at least one knee.

Variable	Value
Total patients	44
Age, years	61.8 ± 8.0
BMI, kg/m²	35.3 ± 5.2
Female sex	30 (68.2%)
Male sex	14 (31.8%)
Riyadh clinic	21 (47.7%)
Jeddah clinic	23 (52.3%)
Bilateral knee OA	28 (63.6%)
Unilateral knee OA	16 (36.4%)
Right-sided OA only	10 (22.7%)
Left-sided OA only	6 (13.6%)
KL grade 2 involvement	15 (34.1%)
KL grade 3 involvement	22 (50.0%)
KL grade 4 involvement	23 (52.3%)
Baseline Lequesne score	19.18 ± 3.41
Baseline VAS pain score	7.48 ± 1.17
Completed six-month follow-up	39 (88.6%)
Lost to six-month follow-up	5 (11.4%)

Before injection, patients had considerable pain and functional limitation, with a mean baseline Lequesne score of 19.18 ± 3.41. At approximately six weeks, the mean Lequesne score decreased to 16.36 ± 3.63. This improvement continued at the six-month assessment, where the mean Lequesne score was 13.28 ± 3.09 among patients with available follow-up data. Paired t-test analysis showed statistically significant improvement in Lequesne scores from baseline to six weeks (t(43) = 32.14, p < 0.001) and from baseline to six months (t(38) = 47.76, p < 0.001). A similar pattern was observed for pain severity, with mean VAS pain score decreasing from 7.48 ± 1.17 at baseline to 5.73 ± 1.53 at six weeks (t(43) = 23.78, p < 0.001) and 4.21 ± 1.20 at six months (t(38) = 45.64, p < 0.001). The changes in Lequesne and VAS scores over time are presented in Table 2.

**Table 2:** Clinical Outcomes Over Time Values are presented as mean ± standard deviation unless otherwise stated. VAS: visual analog scale. Negative mean change indicates improvement from baseline. Six-week analysis included 44 patients, while six-month analysis included 39 patients with available follow-up data. p-values and t-statistics were calculated using paired t-test analysis comparing follow-up scores with baseline values.

Outcome	Baseline	Six Weeks	Six Months	Mean Change at Six Weeks	t-statistic at Six Weeks	Mean Change at Six Months	t-statistic at Six Months	p-value
Lequesne score	19.18 ± 3.41	16.36 ± 3.63	13.28 ± 3.09	-2.82	32.14 (df=43)	-5.51	47.76 (df=38)	<0.001
VAS pain score	7.48 ± 1.17	5.73 ± 1.53	4.21 ± 1.20	-1.75	23.78 (df=43)	-3.15	45.64 (df=38)	<0.001

**Figure 1:**
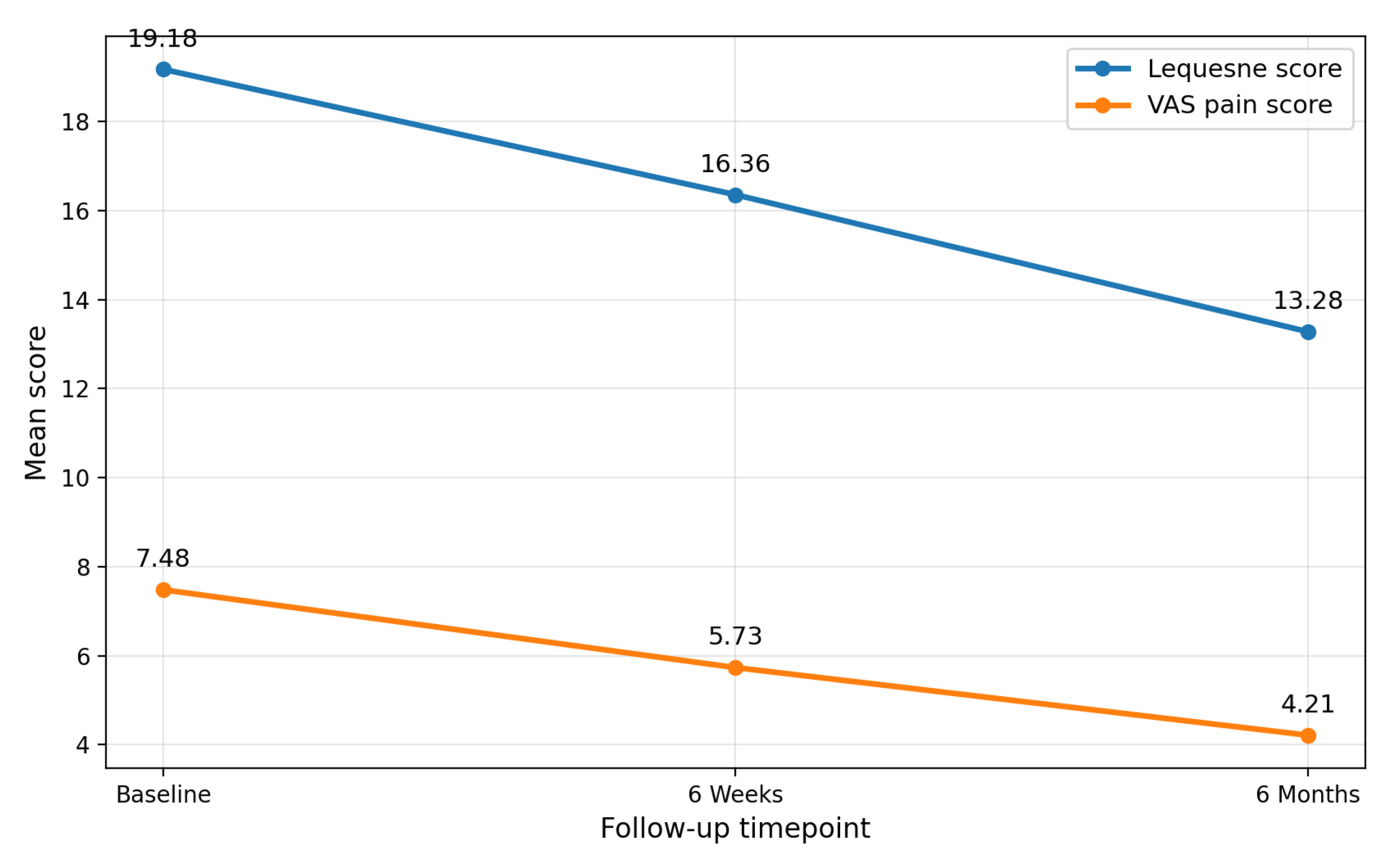
Change in Lequesne and VAS Scores Over Time The figure shows progressive improvement in mean Lequesne and VAS pain scores from baseline to six weeks and six months after injection. VAS: visual analog scale. Lower scores indicate clinical improvement.

The clinical response appeared gradual rather than abrupt. Many patients showed some symptomatic and functional improvement by the six-week visit, with further improvement documented by six months. Patients with more advanced radiographic disease appeared to retain residual symptoms and walking limitation despite improvement from baseline, which is consistent with the clinical pattern commonly seen in advanced knee osteoarthritis. The overall change in Lequesne and VAS scores during follow-up is illustrated in Figure 1.

Thirty-nine patients completed the six-month follow-up, corresponding to a follow-up completion rate of 88.6%. Five patients did not have complete six-month outcome data. Documented reasons included inability to return for follow-up, relocation, and conversion to total knee arthroplasty in one patient with advanced bilateral disease.

A descriptive comparison of patients who completed the six-month follow-up and those lost to the six-month follow-up is shown in Table 3. Patients lost to follow-up were descriptively older, had higher BMIs, and had worse baseline Lequesne and VAS scores. They also more frequently had bilateral OA and KL grade 4 involvement. Because only five patients were lost to six-month follow-up, this comparison was interpreted descriptively, and no formal statistical testing was performed.

**Table 3:** Descriptive Comparison According to Six-Month Follow-Up Completion Values are presented as mean ± standard deviation or number (%). BMI: body mass index; OA: osteoarthritis; KL: Kellgren-Lawrence; VAS: visual analog scale. This comparison was descriptive because of the small number of patients lost to follow-up.

Variable	Completed Six-Month Follow-Up (n=39)	Lost to Six-Month Follow-Up (n=5)
Age, years	60.8 ± 7.7	69.2 ± 6.9
BMI, kg/m²	34.8 ± 5.0	39.6 ± 5.3
Baseline Lequesne score	18.79 ± 3.29	22.20 ± 3.03
Baseline VAS pain score	7.36 ± 1.16	8.40 ± 0.89
Female sex	27 (69.2%)	3 (60.0%)
Bilateral knee OA	24 (61.5%)	4 (80.0%)
KL grade 4 involvement	19 (48.7%)	4 (80.0%)

An exploratory descriptive subgroup analysis was performed according to the worst recorded KL grade per patient among patients who completed six-month follow-up. Patients with KL grade 4 involvement were compared with patients whose worst recorded KL grade was 2 or 3. Both subgroups showed improvement in Lequesne and VAS scores at six months. However, patients with KL grade 4 involvement had higher baseline scores, remained more symptomatic at six months, and reported lower satisfaction. The subgroup analysis is summarized in Table 4.

**Table 4:** Exploratory Descriptive Subgroup Analysis by Worst Recorded KL Grade Values are presented as mean ± standard deviation unless otherwise stated. KL: Kellgren-Lawrence; VAS: visual analog scale. Negative mean change indicates improvement from baseline. This analysis included only patients who completed six-month follow-up and was interpreted descriptively because of the limited sample size.

Subgroup	n	Lequesne Baseline	Lequesne Six Months	Mean Lequesne Change	VAS Baseline	VAS Six Months	Mean VAS Change	Mean Satisfaction
KL grade 4 involvement	19	21.79 ± 1.44	15.95 ± 2.01	-5.84	8.37 ± 0.50	5.16 ± 0.76	-3.21	3.84 ± 0.76
KL grade 2 or 3 involvement	20	15.95 ± 1.47	10.75 ± 1.21	-5.20	6.40 ± 0.68	3.30 ± 0.73	-3.10	4.55 ± 0.51

Patient satisfaction was generally favorable. The mean satisfaction score was 4.21 ± 0.73 out of 5, and most patients reported satisfaction scores of 4 or 5. Lower satisfaction scores were mainly seen in patients who continued to report relevant pain, walking limitation, or difficulty with daily activities at follow-up. Safety outcomes and satisfaction results are summarized in Table 5.

**Table 5:** Safety Outcomes and Patient Satisfaction Values are presented as mean ± standard deviation or number (%). A satisfaction score was available for 39 patients who completed follow-up. Percentages for satisfaction categories were calculated using 39 as the denominator. Percentages for adverse events were calculated using the total cohort of 44 patients as the denominator. No serious treatment-related adverse events or joint infections were documented during the follow-up period.

Variable	Result
Mean satisfaction score	4.21 ± 0.73
Satisfaction score 5/5	14 (35.9%)
Satisfaction score 4/5	20 (51.3%)
Satisfaction score 3/5	4 (10.3%)
Satisfaction score 2/5	1 (2.6%)
Post-injection pain lasting approximately 48 hours	10 (22.7%)
Mild local redness	3 (6.8%)
Mild local swelling	1 (2.3%)
Joint infection	0 (0%)
Serious treatment-related adverse event	0 (0%)
Conversion to total knee arthroplasty during follow-up	1 (2.3%)

The injection was generally well tolerated. The most commonly documented adverse event was short-duration post-injection pain lasting approximately 48 hours. Minor local reactions, including mild redness and swelling, were also recorded in a small number of patients. No joint infection, hospitalization, urgent procedure related to the injection, or other serious treatment-related adverse event was documented during the follow-up period.

## Discussion

The present study reviewed clinical outcomes after a single intra-articular hyaluronic acid-glucosamine injection in patients with symptomatic knee osteoarthritis treated in routine outpatient orthopedic practice. The main finding was that Lequesne Algofunctional Index and VAS pain scores decreased from baseline at approximately six weeks and six months after injection. The improvement was gradual and continued between the early and mid-term follow-up visits. Patient satisfaction was generally favorable, and no serious treatment-related adverse events or joint infections were documented during the follow-up period.

Knee osteoarthritis is not only a radiographic diagnosis. As Hunter and Bierma-Zeinstra emphasized, OA is a major clinical condition associated with pain, disability, and functional decline, particularly in older adults and patients with additional mechanical risk factors [1]. In daily orthopedic practice, patients usually seek help because walking becomes shorter, stairs become harder, sleep may be interrupted by pain, and daily activity becomes less predictable. This practical functional burden was clearly relevant in the present cohort, where many patients had elevated BMI, bilateral disease, and advanced Kellgren-Lawrence grades.

The clinical pathway in this study is consistent with current nonoperative management concepts. Bannuru et al., in the Osteoarthritis Research Society International (OARSI) guideline, described OA management as patient-centered and generally stepwise, including education, exercise-based therapy, weight management, analgesic strategies, and selected procedural options depending on clinical context [2]. The American Academy of Orthopaedic Surgeons (AAOS) nonarthroplasty guideline and the American College of Rheumatology (ACR)/Arthritis Foundation guideline also emphasize structured nonoperative care while acknowledging that treatment decisions are influenced by patient symptoms, comorbidities, and clinical context [3,12]. The patients in this study fit this real-world pathway because they had persistent symptoms despite previous conservative management and were treated in routine outpatient clinics rather than in a highly controlled trial environment.

The Lequesne Algofunctional Index was a suitable primary functional outcome for this retrospective review because it reflects the clinical problems that frequently dominate knee OA consultations: pain or discomfort, maximum walking distance, and activities of daily living. Lequesne and colleagues originally described severity indices for hip and knee OA and reported their value in clinical assessment and follow-up [8]. Faucher et al. later compared the Lequesne and Western Ontario and McMaster Universities Arthritis Index (WOMAC) indexes in knee OA and supported the clinical validity of the Lequesne index as an algofunctional measure [13]. Its practical structure was important in the present study because the score could be derived from routinely documented clinical items without relying on a long questionnaire that may be inconsistently completed in busy outpatient practice.

The use of Lequesne is also supported by cross-cultural and comparative validation work. Xie et al. reported acceptable reliability and validity of Singapore English and Chinese versions of the Lequesne index among Asian patients with knee OA [14]. Basaran et al. compared WOMAC and Lequesne in Turkish patients with hip or knee OA and found both to be clinically useful, although WOMAC performed better in some psychometric domains [15]. These findings are useful for interpreting the present study. The Lequesne index may not be the most detailed OA outcome tool, but it remains a recognized and clinically practical severity/function measure, especially when the goal is to evaluate routine clinical outcomes rather than perform a highly instrumented trial.

The observed Lequesne improvement is clinically coherent. The mean score decreased from 19.18 at baseline to 16.36 at six weeks and 13.28 at six months. Although many patients remained symptomatic at follow-up, the direction and size of change suggest measurable improvement in pain-related function and walking limitation. This is important because the cohort included many patients with advanced structural disease. In such patients, complete resolution after injection therapy would not be expected. A more believable outcome is exactly what was observed here: improvement from baseline, but with residual symptoms in more severe cases. The exploratory subgroup analysis also supports this clinical pattern. Patients with KL grade 4 involvement improved, but they remained more symptomatic at six months and reported lower satisfaction than patients with KL grade 2 or 3 involvement. This is consistent with the broader viscosupplementation literature suggesting that disease severity may influence clinical response [16].

VAS pain scores changed in the same direction as Lequesne scores. Mean VAS decreased from 7.48 at baseline to 5.73 at six weeks and 4.21 at six months. The parallel improvement in pain and algofunctional scores supports the internal consistency of the clinical response. In a retrospective study, agreement between two different outcome measures is useful because it makes the observed improvement less likely to reflect random movement in a single score.

The findings should be interpreted within the broader and still debated literature on intra-articular hyaluronic acid. Jüni et al. evaluated intra-articular hylan or hyaluronic acid preparations in knee OA and contributed to the evidence base showing that response may vary across products and patient groups [17]. Petrella and Petrella reported improvement after intra-articular hyaluronic acid in a randomized placebo-controlled study [18]. Strand et al. and Miller and Block also reported favorable findings for US-approved viscosupplements in systematic reviews and meta-analyses of randomized saline-controlled trials [19,20]. More recently, Migliorini et al. reported that intra-articular hyaluronic acid injections may reduce pain compared with placebo in knee osteoarthritis [5]. These studies support the plausibility of symptomatic improvement after viscosupplementation.

At the same time, the evidence is not uniformly positive. Rutjes et al. raised concerns about the clinical relevance and safety profile of viscosupplementation in a systematic review and meta-analysis [21]. Pereira et al. reported that viscosupplementation was associated with only a small reduction in pain compared with placebo in a large BMJ systematic review [4]. Bannuru et al. also examined comparative safety across hyaluronic acid products, emphasizing that safety profiles and product characteristics may differ [22]. Tschopp et al. compared glucocorticoid, hyaluronic acid, platelet-rich plasma, and placebo injection arms in knee OA, highlighting the importance of comparator groups when interpreting injection outcomes [23]. This mixed literature is important. The present study should therefore be interpreted as a real-world clinical outcomes review, not as proof of superiority, disease modification, cartilage restoration, or independent product-specific efficacy.

The timing of improvement is also clinically relevant. He et al. reported in a meta-analysis that corticosteroid injections may provide stronger short-term pain relief, while hyaluronic acid may show relatively better effects at later follow-up points [24]. In the present study, improvement was already seen by approximately six weeks and continued at six months. This pattern is compatible with the delayed or progressive response often discussed in viscosupplementation studies. However, because the current study had no control group, it is not possible to separate the injection effect from natural symptom fluctuation, physiotherapy, analgesic use, activity modification, contextual effects, or patient expectation.

The glucosamine component deserves careful interpretation. Most glucosamine evidence in OA relates to oral glucosamine or oral glucosamine-chondroitin combinations, not injectable intra-articular combination products. Wandel et al. reported limited overall benefit for glucosamine or chondroitin in a network meta-analysis [6]. A more recent systematic review by Vo et al. summarized evidence on the effectiveness and safety of glucosamine in osteoarthritis, mainly in relation to oral glucosamine preparations [7]. Towheed et al. reviewed glucosamine therapy for osteoarthritis in the Cochrane Database and also showed that conclusions vary depending on study quality, preparation, and outcome assessment [25]. Clegg et al., in the GAIT trial, found no significant benefit in the overall study population, although subgroup findings have often been discussed [26]. In contrast, Reginster et al. reported favorable long-term findings with glucosamine sulfate in a randomized trial [27].

A biological rationale for N-acetylglucosamine can be considered because glucosamine derivatives are related to glycosaminoglycan and cartilage matrix pathways, and experimental chondrocyte work has evaluated metabolic effects of glucosamine and N-acetylglucosamine in human articular chondrocytes [28]. However, such mechanistic data cannot be directly translated into clinical efficacy. The oral glucosamine literature also cannot be directly transferred to the injectable preparation used here. Therefore, this study reports the clinical outcomes of a hyaluronic acid-based combination injection and does not prove that N-acetylglucosamine independently caused the observed improvement.

The safety findings were reassuring. The most common documented adverse event was short-duration post-injection pain, while mild redness and swelling were uncommon. No septic arthritis, hospitalization, urgent procedure, or serious treatment-related adverse event was recorded. This is broadly consistent with published safety analyses of intra-articular hyaluronic acid products, including the systematic review by Miller et al. involving more than 8,000 patients [29]. Nevertheless, retrospective safety data must always be interpreted with caution, because minor self-limited events may be underreported in routine clinical records.

One strength of this study is that it reflects routine outpatient orthopedic practice. Patients with obesity, bilateral symptoms, and advanced radiographic OA were not excluded, and the follow-up windows were practical rather than artificially rigid. This matters because many trials include more controlled populations and stricter follow-up schedules than those seen in daily clinical work. The six-month follow-up completion rate of 88.6% is also reasonable for a retrospective clinical review.

Another strength is the use of an algofunctional index that is short and clinically direct. In a busy clinic, lengthy questionnaires may not always be completed consistently, especially when patients are elderly, symptomatic, or attending for routine follow-up. The Lequesne index allowed assessment of pain and function in a way that remained close to the actual clinical conversation: pain, walking distance, stairs, and daily activity. This practical feature is particularly relevant to the retrospective nature of the study.

This study has several limitations. First, it was retrospective and included a relatively small sample. Second, there was no untreated control group, hyaluronic-acid-only arm, corticosteroid comparator arm, or placebo injection arm. Intra-articular injection studies may show clinically important placebo and contextual responses, and the present design cannot separate the effect of the injection from natural symptom fluctuation, physiotherapy adherence, analgesic use, activity modification, weight change, or patient expectations [30]. Third, outcomes were analyzed at the patient level rather than the knee level. This approach reflects routine clinical documentation, but bilateral OA may influence patient-reported Lequesne and VAS scores and may reduce the precision of knee-specific interpretation. Fourth, five patients were lost to the six-month follow-up. These patients were descriptively older, had higher BMIs, had worse baseline scores, and more often had KL grade 4 involvement, which may have biased the six-month results toward better-observed outcomes among completers. Finally, follow-up was limited to six months, so longer-term durability and any effect on future arthroplasty timing cannot be determined.

The study provides useful preliminary real-world information on a hyaluronic acid-glucosamine combination injection in symptomatic knee osteoarthritis. The findings suggest improvement in pain and algofunctional outcomes over six months, with favorable patient satisfaction and no serious safety signal in this cohort. Larger prospective comparative studies are needed to determine whether this combination provides additional benefit over hyaluronic acid alone, corticosteroid injection, or other nonoperative treatment pathways.

## Conclusions

In this retrospective multicenter cohort, a single intra-articular hyaluronic acid-glucosamine injection was associated with improvement in the Lequesne Algofunctional Index and VAS pain scores over a six-month follow-up period in patients with symptomatic knee osteoarthritis. Patient satisfaction was generally favorable, and no serious treatment-related adverse events or joint infections were documented.

These findings suggest that this injectable combination may be a clinically practical and generally well-tolerated option in routine outpatient care. However, the results should be interpreted cautiously because of the retrospective design, small sample size, loss to follow-up, patient-level outcome analysis, and absence of a comparator group. Larger prospective comparative studies are needed to clarify the durability of response and determine whether the combination provides additional benefit over hyaluronic acid alone or other nonoperative injection strategies.
